# Cancer screening in Portugal: sex differences in prevalence, awareness of organized programmes and perception of benefits and adverse effects

**DOI:** 10.1111/hex.12450

**Published:** 2016-02-23

**Authors:** Ana Rute Costa, Susana Silva, Pedro Moura‐Ferreira, Manuel Villaverde‐Cabral, Osvaldo Santos, Isabel do Carmo, Henrique Barros, Nuno Lunet

**Affiliations:** ^1^ Department of Clinical EpidemiologyPredictive Medicine and Public HealthUniversity of Porto Medical SchoolPortoPortugal; ^2^ EPIUnitInstitute of Public HealthUniversity of Porto (ISPUP)PortoPortugal; ^3^ Institute of Social SciencesUniversity of LisbonLisbonPortugal; ^4^ Institute of Preventive Medicine and Public HealthFaculty of Medicine of LisbonLisbonPortugal; ^5^ Faculty of MedicineUniversity of LisbonLisbonPortugal

**Keywords:** early detection of cancer, health knowledge, attitudes, practice, neoplasm

## Abstract

**Background:**

Cancer screening has contributed to downward trends in cancer mortality, but is also associated with adverse effects, which highlights the importance of promoting the participation based on informed decisions.

**Objectives:**

We aimed to describe the use of cancer screening (either in organized programmes or as opportunistic screening), awareness of organized programmes and perception of its potential benefits and adverse effects, depicting possible sex differences.

**Design and methods:**

We evaluated 1624 Portuguese‐speaking dwellers, aged between 16 and 79 years, through face‐to‐face interviews. To quantify sex differences, adjusted prevalence ratios and respective 95% confidence intervals were computed using Poisson regression.

**Results:**

Among eligible age groups, the lifetime prevalence of screening for breast and cervical cancers was 89.8 and 71.9%, respectively. The prevalence was 23.7% for colorectal cancer and no significant sex differences were observed. Prostate cancer screening was reported by 63.8% of men. Over half of the participants referred that cancers such as prostate, skin, lung and stomach should be screened for, in addition to those for which organized programmes are recommended. Reassurance by negative results was identified as the main potential benefit of screening by nearly one‐third of men and women. Anxiety while waiting for results was the most mentioned potential adverse effect (60.4%); men refer less often this and financial costs, although statistical significance of these results was borderline.

**Conclusions:**

This study provides a benchmark to plan and monitor the effects of awareness‐raising interventions, as well as for international comparisons across countries with different cancer prevention and control structures.

## Introduction

In the last few decades, an increasing number of deaths due to oncologic diseases has been observed worldwide,^1^ and almost 15 million cancer deaths are expected by 2035.^2^ In Portugal, just over 24 000 deaths were observed in 2012,^2^ and, among men, cancer already surpassed cardiovascular disease as the leading cause of years of life lost.^3^ Additionally, for the next decades, it is expected a 38% increment in the overall number of deaths attributable to cancer in Portuguese population, with a higher increase in men than in women (41% vs. 33%),^2^ if the trends in mortality observed in the last few years are maintained.

Regardless of the increasing absolute number of deaths due to cancer, which can be partially explained by population growth and ageing,^4^ in high‐income countries, a downward trend in overall cancer age‐standardized mortality rates has been recently observed.^1^ This is likely to reflect decreases in the incidence of tobacco‐related cancers,^5^ and the access to more effective treatment and improvements on earlier detection of cancer.^6^ Nevertheless, cancer screening is associated with overdiagnosis and overtreatment,^7^ among other potential adverse effects, which highlights the importance of promoting informed decisions regarding the participation in cancer screening programmes.

Cancer screening can be performed as part of organized programmes or opportunistically. The former requires a documented policy where the targeted population groups, the screening tests and screening intervals are defined and implies a team and an administrative structure responsible for inviting the eligible population, service delivery, quality assurance and evaluation at the national or regional level. The latter corresponds to screening conducted unsystematically, taking advantage of the contact of the potentially eligible subjects with the health system.^8^, ^9^

According to the Council of the European Union,^10^ organized programmes for cancer screening are recommended for breast, cervical and colorectal cancers. In Portugal, screening programmes are implemented by each of the regional health administrations; although the potentially eligible subjects are identified through the primary health‐care centres and invited to participate in the screening programmes for these cancers, there are differences in the management, population coverage and date of onset according to region and type of cancer.

Organized screening for breast cancer was started in 1990 and has been gradually expanding to the entire country; in 2012, it was estimated that approximately two‐thirds of the population was covered. In most regions, the organized screening targets women aged 45–69 years, to be screened by mammography every 2 years.^11^

For cervical cancer, organized screening was first implemented in 1990; in 2012, just over 40% of the eligible population was covered. In most regions, organized screening targets women aged 25–64 years, to be screened by conventional cytology (Pap test) or liquid‐based cytology; complementary testing for human papillomavirus (HPV) infection is available in some regions.^11^ In a clinical norm for diagnosis and staging of cervical cancer, the Portuguese Directorate‐General of Health recommends that screening for this type of cancer should be provided to all women between 25 and 64 years of age that were not previously screened.^12^

Organized screening for colorectal cancer started in 2009; in 2012, only two regions of mainland Portugal had implemented such programmes, which translate into 9.3% of the country's eligible population being covered. Organized colorectal cancer screening targets men and women aged between 50 and 69 or 70 years, to be tested by a faecal occult blood test (FOBT) every 2 years.^11^ In addition, to increase the opportunistic screening, the Portuguese Directorate‐General of Health recommends annual prescription of FOBT in all asymptomatic individuals aged between 50 and 74 years.^13^

According to guidelines recently issued by the Portuguese Directorate‐General of Health, opportunistic screening using prostate‐specific antigen's evaluation (PSA) may be prescribed to men who request this examination, after them being informed about the potential benefits and risks associated with this procedure.^14^

In addition to distinct cancer screening recommendations for men and women, sex differences regarding cancer knowledge and use of screening were previously outlined. Women are commonly described as having higher levels of cancer awareness,^15^ providing higher estimates of lifetime risk of cancer,^16^ and are more worried in getting cancer than men.^17^ However, concerning the adherence to cancer screening, the results are less consistent, and some studies show that men may attend cancer screening more frequently than women, depending on the type of examination.^18^ Sex differences regarding the perception of facilitators and barriers associated with cancer screening uptake have also been observed.^19^, ^20^

Therefore, this study aims to describe the use of cancer screening, awareness of organized programmes and perception of its potential benefits and adverse effects, depicting possible differences between men and women.

## Methods

### Study population

The present analysis was based on a national survey conducted in 2012, aiming to assess knowledge and health behaviours of the Portuguese population aged between 16 and 79 years.

The study selected a representative sample of Portuguese‐speaking dwellers in mainland Portugal, using a multistage sampling design, defined according to results of the 2001 Portuguese Census.^21^ A probabilistic sampling procedure, stratified by NUTS II – Territorial Nomenclature Units for Statistical Purposes, level II (North, Centre, Lisbon and Tagus Valley, *Alentejo* and *Algarve*) and by the number of inhabitants in geographical units with at least 10 dwellings (<2000, 2000–9999, 10 000–19 999, 20 000–100 000, >100 000), was used to select 150 geographical units, among which a total of 585 starting points were designated for the selection of households through standard random route procedures. All of the potentially eligible dwellers were identified in each selected household, and only the one whose previous birthday was closest to the date of the contact was invited to participate; a total of 1624 valid interviews were obtained (response rate: 70.8%).

### Data collection

Participants were evaluated through face‐to‐face interviews, using a structured questionnaire.

The questions regarding the use of screening were preceded by a brief explanation highlighting that the questions refer to tests performed in the absence of disease symptoms, that is, have been carried out routinely aiming to early detection. The use of cancer screening at least once during the participants’ lives was assessed for breast (among women aged ≥30 years: *Did you ever perform a mammography testing for breast cancer screening?*), cervix (among all women: *Did you ever use cervical cancer screening, i.e. the Pap smear test, on a routine basis?*), colon and rectum (among women and men aged ≥40 years: *Did you ever perform a FOBT for colorectal cancer screening, on a routine basis?; Did you ever perform a colonoscopy for colorectal cancer screening, on a routine basis?*), and prostate [among men aged ≥40 years: *Did you ever use prostate‐specific antigen (PSA) testing (blood analysis for prostate cancer screening), on a routine basis?; Did you ever use prostate cancer screening, i.e. digital rectal examination (DRE), on a routine basis?*]. To estimate the lifetime prevalence of use of screening for each of these cancers, we considered only the age groups that should be screened for, according to the screening policy adopted in Portugal: breast – 45–69 years^11^; cervix – 25–64 years^11^, ^12^; colorectal – 50–74 years^11^, ^13^; prostate – 55–69 years.^14^

Participants were also asked to indicate whether specific cancers should be screened for after a certain age, including those with national programmes, such as breast, cervical and colorectal, and other frequent cancers for which there is no organized screening, namely prostate, stomach, lung and skin (*Which of the following cancers should be screened for, after a certain age?* – for each cancer, possible answers were ‘yes’, ‘no’, ‘do not know’ and ‘did not answer’; for data analysis, the option ‘do not know’ and ‘did not answer’ was coded as ‘no’).

The perception of potential benefits of cancer screening was evaluated through selection of the main benefit (*Which is the main benefit of participating in cancer screenings?),* from the following list: ‘earlier detection’, ‘more effective treatment’ and ‘knowledge of not having the disease’. For several potential adverse effects of screening, participants were asked to indicate whether they fear that these problems may occur after participation in cancer screening (*Do you fear that each of the following effects may occur after a cancer screening?* – possible answers were ‘yes’, ‘no’, ‘do not know’ and ‘did not answer’; for data analysis, the option ‘do not know’ and ‘did not answer’ was coded as ‘no’), namely ‘pain or discomfort due to medical examinations’, ‘being anxious while waiting for the results’, ‘the test shows that you are ill when you are not’, ‘the test does not show that you are ill when you actually are’, ‘undergoing unnecessary treatments’, ‘anticipated diagnosis of an incurable disease’ and the ‘financial costs for yourself (e.g. travel expenses)’.

### Statistical analysis

Statistical analyses were performed using STATA^®^ version 11.1 (College Station, TX, USA, 2009). To estimate the associations between sex and use, knowledge and perceptions about cancer screening, we computed adjusted prevalence ratios (PR) and respective 95% confidence intervals (95% CI) using Poisson regression models, including age and educational level. The product of design and population weights was also computed and used in all analyses; the former were used to compensate for the unequal probability of selection, and the latter, to correct the discrepancy between sample composition and the Portuguese population regarding sex, age, education, marital status and NUTS II distribution and size of the geographical units.

### Ethical approval

This survey was approved by the Ethics Committee of the University of Porto (33/CEUP/2012), and all participants provided written informed consent.

## Results

The sample included a similar proportion of men and women, mostly from the North and South regions of mainland Portugal. Subjects younger than 30 years corresponded to less than 30% of the sample, with a higher proportion among men, and participants aged 70–79 years were nearly 9%, with a higher proportion among women. Just over 45% of women and nearly 40% of men had 0–4 years of schooling, whereas the proportion of those with more than 12 years of schooling was higher among women (16.0% vs. 11.7%) (Table 1).

**Table 1:** Characteristics of participants (*n* = 1624)

	Women		Men	
	Non‐weighted %	Weighted %	Non‐weighted %	Weighted %
All participants	61.4	50.3	38.6	49.7
Region of residence (NUTS II)^a^				
North	40.7	40.4	39.6	39.9
Centre	23.6	18.3	18.8	17.4
South^b^	35.8	41.3	41.5	42.6
Age (years)				
<30	11.7	25.4	18.5	29.9
30–39	12.8	15.5	15.3	17.8
40–49	14.6	19.5	14.4	18.7
50–59	18.5	15.3	15.6	13.4
60–69	23.2	13.4	20.3	12.4
70–79	19.1	10.8	15.8	7.8
Education (years)				
0–4	53.2	45.3	42.0	39.7
5–9	16.0	14.6	22.5	22.2
10–12	17.4	24.0	22.4	26.4
>12	13.4	16.0	13.1	11.7

a NUTS II, Territorial Nomenclature Units for Statistical Purposes, level II.

b Includes the regions of Lisbon and Tagus Valley, *Alentejo* and *Algarve*.

Percentages may not total 100 due to rounding.

**Figure 1:**
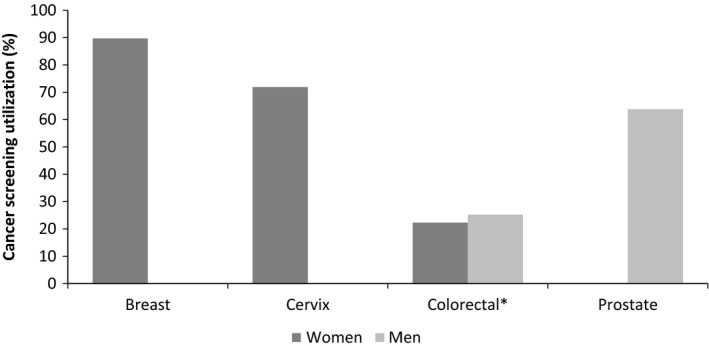
Prevalence of cancer screening utilization during lifetime, among Portuguese women and men. *Age and education‐adjusted prevalence ratio (men vs. women) = 1.14 (95% CI: 0.81–1.59). Use of cancer screening was only considered among the following age groups: *Breast* (mammography) – 45–69 years of age (only women)^11^; *Cervix* (cervical cytology testing) – 25–64 years of age (only women)^11^, ^12^; *Colorectal* (faecal occult blood test and/or colonoscopy) – 50–74 years of age (all)^11^, ^13^; *Prostate* (prostate‐specific antigen and/or digital rectal examination) – 55–69 years of age (only men).^14^

Regarding the lifetime prevalence of cancer screening among eligible age groups, 89.8% (95% CI: 85.7–93.8) of women between 45 and 69 years reported a previous use of mammography and, among women aged 25–64 years, 71.9% (95% CI: 66.5–77.3) had a screening cervical cytology before. Regarding colorectal cancer screening, the lifetime prevalence among participants aged 50–74 years was 23.7% (95% CI: 19.4–27.9), with no statistically significant sex differences (PR = 1.14; 95% CI: 0.81–1.59). Among men aged 55–69 years, 63.8% (95% CI: 54.9–72.8) reported to have been screened for prostate cancer (Fig. 1).

**Figure 2:**
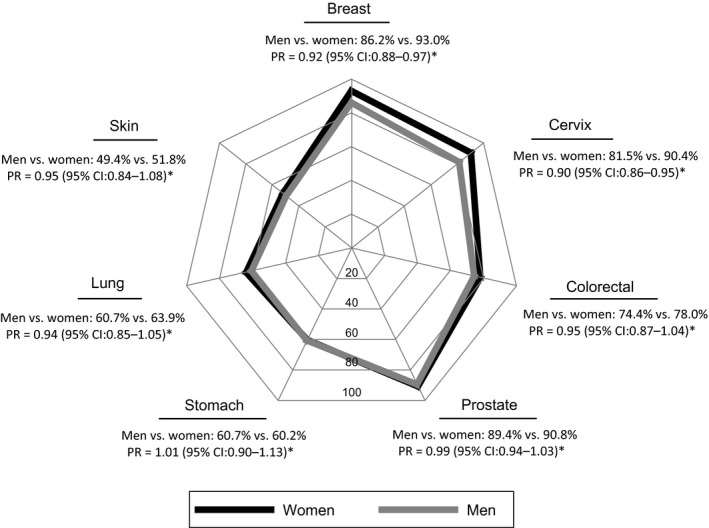
Proportion of Portuguese women and men that identified cancers for which screening should be warranted. CI, confidence interval; PR, prevalence ratio. *Adjusted for age and education level.

Approximately 90% of participants considered that screening was recommended for prostate, breast and cervix cancers (90.1, 89.6 and 86.0%, respectively), and a lower proportion of the participants identified colorectal (76.2%), lung (62.3%), stomach (60.5%) or skin (50.6%) cancers, as conditions that should be screened for after a specific age. There were no statistically significant sex differences, except for men reporting less frequently that screening for breast (PR = 0.92; 95% CI: 0.88–0.97) and cervix (PR = 0.90; 95% CI: 0.86–0.95) cancers should be performed after a certain age (Fig. 2).

Concerning the potential benefit of cancer screening, a higher proportion of respondents identified the early detection of cancer as the main advantage (58.6%), followed by the reassurance by negative results (31.4%) and more effective treatment (10.0%), with no significant statistical difference between sexes. The identification of potential adverse effects of cancer screening ranged between 41.0%, for overtreatment, and 60.4%, for anxiety while waiting for results. Men were less likely to refer all potential adverse effects, particularly anxiety while waiting for results (PR = 0.89; 95% CI: 0.79–1.00) and financial costs (PR = 0.86; 95% CI: 0.74–1.00), although the statistical significance of these results was borderline (Table 2).

**Table 2:** Perception of potential benefits and adverse effects of cancer screening according to sex

	Proportion of participants identifying each potential benefit or adverse effect of cancer screening (%)			PR (95% CI)^a^
	All	Women	Men	
Potential benefits				
Earlier detection or more effective treatment^b^^,^ c	68.6	68.0	69.3	1.02 (0.92–1.12)
Potential adverse effects				
Anxiety while waiting for results	60.4	63.9	56.9	0.89 (0.79–1.00)
Anticipated diagnosis	52.1	53.9	50.3	0.93 (0.81–1.07)
Pain or discomfort	51.9	52.7	51.2	0.97 (0.85–1.11)
Financial costs	44.9	48.1	41.7	0.86 (0.74–1.00)
False positives	43.6	47.1	40.0	0.85 (0.72–1.01)
False negatives	42.7	46.3	38.9	0.85 (0.71–1.02)
Overtreatment	41.0	41.9	40.2	0.97 (0.82–1.15)

CI, confidence interval; PR, prevalence ratio.

a Adjusted for age and education level.

b vs. knowledge of not having the disease.

c Excluding 2.5% of participants that did not know/answer.

## Discussion

The lifetime prevalence of screening varied widely between the three cancers with organized screening programmes, which are likely to reflect the marked asymmetries in their population coverage across regions and time of implementation, as well as inequalities in the access to opportunistic screening. Organized programmes for breast and cervical cancers screening were the first to be implemented in Portugal, but they still are not available for all the eligible population.^11^ Nevertheless, two previous studies showed high prevalence of screening in regions with no organized programme for breast 22 or cervical cancer,^23^ which may reflect an easy access to opportunistic screening in these regions. Although the early detection of cancer by female breast mammography and cervical cytology testing contribute to an overall higher frequency of screening among women, this does not reflect inequalities in the access to screening according to sex because these cancers affect only women.

Organized screening programmes for colorectal cancer have only recently begun, and, in 2012, less than 10% of the population was covered.^11^ Furthermore, examinations for screening colorectal cancer, specially colonoscopy, are frequently perceived as painful, leading to anxiety, discomfort, feelings of vulnerability and embarrassment.^24^ These facts may contribute to explain the low prevalence of this type of screening observed in our study, despite the high proportion of participants mentioning colorectal as a cancer that should be screening for.

Although population‐based prostate cancer screening is not recommended, the high prevalence of this cancer,^2^ with survivors sharing their experience with their social network, and the focus given by mass‐media to prostate cancer,^25^ may contribute to our observation of nearly two‐thirds of men reporting to have undergone opportunistic prostate cancer screening. This prevalence is close to that of women being screened for cervix cancer and is much higher than the lifetime prevalence of colorectal cancer among men and women, in the corresponding eligible age groups.

In the present study, even cancers for which screening is not recommended, including prostate or lung, were frequently mentioned as cancers that should be screened for, drawing attention to patients’ educational and informational needs. Previous studies demonstrated a general population′s enthusiasm regarding cancer screening 26 and a trend towards increasing use of sophisticated technologies for detection of asymptomatic diseases at early stages.^27^ The latter finding may also underlie the recognition of early detection as the main advantage of screening, both among men and women. The fact that we did not find sex differences regarding the benefits of cancer screening is also in accordance with prior findings, showing that men and women are aligned concerning the benefits but not in relation to the barriers for screening uptake.^19^ On the other hand, one‐third of participants valued the fact that screening may allow the reassurance of negative results, which needs to be taken into account when providing balanced information regarding the benefits and harms that may be expected from screening by the potential participants.

Screening for oncologic diseases is not void of risks, including overdiagnosis and overtreatment.^7^ However, these adverse effects were the least reported by our participants. In fact, overdiagnosis is a concept difficult to understand, and its acceptability is variable according to the type of cancer, people's age and educational level, but a previous study did not show differences between men and women.^28^ Additionally, information concerning cancer screenings available in magazine articles, websites and information brochures used for cancer screening invitation is most of the times incorrect or incomplete, and the benefits of screening are over‐reported compared with the harms.^25^, ^29^, ^30^ The fact that anxiety while waiting for the results was the main reported disadvantage of cancer screening may also reflect the societal risk portfolio that rank threats and harms hierarchically taking into account the cultural norms.^31^ In fact, there is a cultural perception of cancer as a disease that is incurable, painful and with difficult treatment,^32^ which may cause anxiety for the screening results. This finding also highlights the importance of defining a short waiting time between the different steps of screening as indicators to be evaluated in organized programmes.

Furthermore, it was previously reported that women have more medical visits,^33^ as well as higher levels of cancer awareness,^15^ perception of lifetime risk of cancer 16 and cancer worry.^17^ These findings, in addition to the highest coverage of national screening programmes targeting only women, may contribute to higher levels of perceived adverse effects of cancer screening found among them, particularly anxiety and financial costs, although the associations observed were not statistically significant. Previous studies have also shown that men tended to underreport medical and psychological symptoms such as pain and emotional distress focusing predominantly on the external and material consequences of diseases 34 and that may be willing to receive less information on cancer screening than women.^20^

This study was based on a representative sample of Portuguese population, evaluated using standardized methods. Nevertheless, there are some limitations that should be highlighted. The data for the present analysis were self‐reported, which may have contributed for an overestimation of the use of cancer screening. According to prior reports, the estimates retrieved from self‐report data are frequently inflated,^35^ probably due to recall, acquiescence or social desirability biases,^36^, ^37^ as well as difficulties in distinguishing between medical exams performed for cancer screening and those for diagnosis and surveillance. Additionally, it is not possible to distinguish cancer screening performed within an organized programme or opportunistic screening.

The high proportion of subjects identifying the cancers that should be screened for, including prostate cancer, for which no organized screening is warranted, as well as potential adverse effects of cancer screening being selected by most participants, may be related with the use of prompted questions. In fact, a previous study has shown that this type of question is associated with higher cancer‐specific knowledge when compared with unprompted (recall) conditions.^38^

Our study is also limited by its cross‐sectional design, as health‐related knowledge can be associated with adherence to screening, but participation in cancer screening can also act as a ‘teachable moment’, contributing to improve knowledge on cancer screening, as well as to modulate the perception of its beneficial and adverse effects.

The external validity of our findings may be limited to some extent due to the specificities of the Portuguese setting regarding the universal coverage of the National Health Service, financed essentially by taxes. This may contribute to reduce inequalities in the access to health care, even when organized screening is not reaching the whole eligible population, in addition to the local cultural norms that may influence the use of cancer screening by men and women. However, our results provide a benchmark for comparisons regarding overall use of screening, as well as sex differences, across countries with distinct types of health‐care systems and cultural specificities, and at different stages of implementation of organized screening,^39^ that may bring them closer or more distant from the Portuguese population. Such comparisons may contribute to understand the potential impact of the implementation of organized screening in the prevention and control of cancer, in reducing inequalities and in the efficient use of resources, taking into account that it may depend on the extent to which opportunistic screening is already taking place.

## Conclusions

The present study shows no substantial sex differences in the use of screening, except for those resulting from the fact that breast and cervical cancer screening apply only to women and prostate only to men, or regarding the potential benefits and adverse effects of screening. It provides a benchmark to plan and monitor the effects of awareness‐raising interventions, as well as for international comparisons across countries with different levels of implementation of cancer prevention and control structures.

## Conflict interest statement

The authors declare that there are no conflicts of interest.

## Source of funding

This work was supported by a grant from *Fundação para a Ciência e a Tecnologia* (HMSP‐IISE/SAU‐ICT/0004/2009).
